# Macrophage‐derived reactive oxygen species promote *Salmonella* aggresome formation contributing to bacterial antibiotic persistence

**DOI:** 10.1002/imt2.70059

**Published:** 2025-06-22

**Authors:** Xiao Chen, Kefan Fang, Bo Li, Yingxing Li, Yuehua Ke, Weixin Ke, Tian Tian, Yifan Zhao, Linqi Wang, Jing Geng, Mark C. Leake, Fan Bai

**Affiliations:** ^1^ Biomedical Pioneering Innovation Center (BIOPIC), Peking‐Tsinghua Center for Life Sciences, School of Life Sciences Peking University Beijing China; ^2^ Medical Research Center, State Key Laboratory of Complex Severe and Rare Diseases, Peking Union Medical College Hospital Chinese Academy of Medical Science Beijing China; ^3^ Chinese PLA Center for Disease Control and Prevention Beijing China; ^4^ State Key Laboratory of Mycology, Institute of Microbiology Chinese Academy of Sciences Beijing China; ^5^ College of Life Sciences University of Chinese Academy of Sciences Beijing China; ^6^ National Local Joint Engineering Research Center of Biodiagnostics and Biotherapy The Second Affiliated Hospital of Xi’ an Jiaotong University Xi’ an China; ^7^ School of Physics, Engineering and Technology University of York York UK; ^8^ Department of Biology University of York York UK

## Abstract

In this study, we reveal that macrophage‐derived reactive oxygen species (ROS) can trigger the rapid formation of *Salmonella* aggresomes, which substantially contribute to the increased frequency of persisters induced by phagocytosis. *Salmonella* containing aggresomes exhibited a dormant phenotype characterized by reduced adenosine triphosphate (ATP) levels and decreased metabolic activity. Furthermore, these dormant bacteria showed upregulated expression of *Salmonella* pathogenicity island 1 (SPI‐1)‐encoded type III secretion system (T3SS)‐related genes, followed by later expression of SPI‐2 T3SS‐related genes when macrophages ROS production declined. Our results demonstrate that *Salmonella* containing aggresomes can enter a dormant state to escape antibiotic attack, while crucially maintaining the ability to resuscitate when the stress environment is improved. Research on bacterial aggresomes could potentially provide therapeutic strategies to combat bacterial antibiotic persistence.

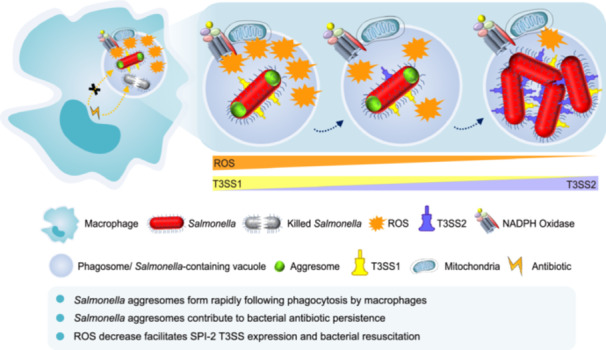

## ETHICS STATEMENT

The ethics application was approved by the Research Ethics Committee of the Institute of Microbiology, Chinese Academy of Sciences, Beijing (No. SQIMCAS2020148).

To the Editor,

Bacterial persisters represent a subpopulation of phenotypic variants that survive antibiotic attack and subsequently resuscitate [1], serving as reservoirs for recurrent infections [2]. Bacterial dormancy is regarded as a prominent theory in elucidating persister formation, as dormant bacteria show decreased metabolic activity and suppressed proliferation rates [2, 3]. Several molecular mechanisms are involved in bacterial dormancy [4]. For example, reactive oxygen species (ROS) deactivate the tricarboxylic acid (TCA) cycle, resulting in a reduction in respiration and adenosine triphosphate (ATP) generation [5]. In addition, the activation of type I toxin TisB and HokB causes ATP leakage, leading to cell dormancy [6]. The type II toxin HipA triggers cell dormancy by phosphorylating the essential translation factor EF‐Tu [7].

Upon host entry, pathogenic bacteria encounter multiple stresses including acidic pH, nutrient limitation, ROS, and reactive nitrogen species [8]. A previous study reported that following macrophage phagocytosis, the proportion of *Salmonella* persisters can greatly increase depending on the expression of several type II toxin‐antitoxin (TA) genes induced by vacuolar acidification and (p)ppGpp synthesis [9]. However, several independent follow‐up studies have argued that deletion of 10 type II TA modules does not affect persister cell levels [10, 11]. Moreover, type II toxins are not always activated under diverse stress conditions [11]. Therefore, the critical mechanism mediating macrophage‐induced antibiotic persistence remains unclear.

Recently, several studies have elucidated the correlation between bacterial aggresomes and dormancy [3, 12, 13]. Bacterial aggresomes contain many proteins that are vital for cellular functions associated with carbon metabolism, oxidative phosphorylation, transcription, and translation [3]. The formation of bacterial aggresomes is driven by liquid–liquid phase separation [12]. Here, we explore the relationship between aggresomes formation and macrophage‐induced bacterial antibiotic persistence in *Salmonella*.

## RESULTS AND DISCUSSION

### *Salmonella* aggresomes form rapidly following phagocytosis by macrophages

**Figure 1:**
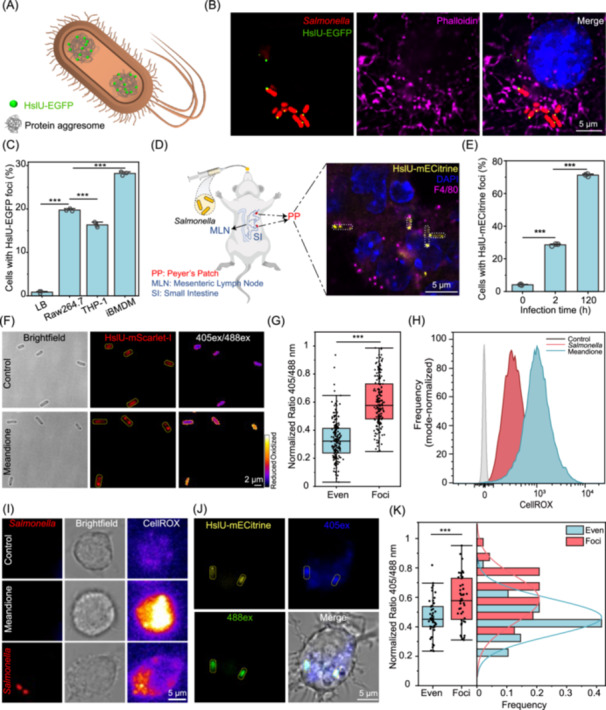
The presence of *Salmonella* aggresomes within macrophages. (A) Schematic of fluorescently labeled bacteria. (B) Immunofluorescence microscopy imaging of bacterial aggresomes within macrophages. The actin cytoskeleton was stained with phalloidin (magenta), and nuclei were stained with DAPI (blue) (scale bar, 5 μm). (C) Percentage of *Salmonella* in the stationary phase possessing HslU‐EGFP foci after internalization by RAW264.7 macrophages, THP‐1 cells, and iBMDMs. Each data point represented an independent biological replicate (*n* = 3); 200 bacteria were analyzed per replicate. (D) Representative confocal micrograph of *Salmonella* aggresomes in infected murine PPs at 120 h.p.i. The cryosection was labeled with an antibody to the macrophage marker F4/80 (magenta). Aggresomes were labeled by HslU‐mECitrine (yellow) (scale bar, 5 μm). (E) Percentage of *Salmonella* cells with HslU‐mECitrine foci in murine PPs at 2 and 120 h.p.i.; *n* = 3. (F) Images of *Salmonella* showing aggresomes and ROS levels following challenge with 160 μM menadione. Bacterial aggresomes were labeled with HslU‐mScarlet‐I, and the 405ex/488ex ratio was used as a measurement of roGFP2_oxidized_/roGFP2_reduced_ (scale bar, 2 μm). (G) Average ROS levels of bacteria with and without aggresomes. (H, I) *Salmonella* infection promoted ROS production in macrophages. (H) Representative histograms of CellROX. (I) Fluorescence microscopy images of ROS production in macrophages. *Salmonella* were labeled with mCherry (scale bar, 5 μm). (J) Live‐cell imaging of bacterial ROS levels and aggresome formation in *Salmonella* within RAW264.7 cells at 1.5 h.p.i. (scale bar, 5 μm). (K) Histogram of the ROS levels within intracellular bacteria with and without aggresomes. (C) and (E) were assessed using one‐way ANOVA followed by Bonferroni post hoc test; (G) and (K) were assessed using two‐tailed unpaired *t*‐test. Error bars indicate standard deviation; *p < 0.05, **p < 0.01, and ***p < 0.001.

*Salmonella Typhimurium* (*S*. *Typhimurium*) SL1344 HslU was genetically fused with EGFP to enable visualization of bacterial protein aggresomes (Figure 1A, Figure S1A,B). Following phagocytosis of fluorescently labeled *Salmonella* at a multiplicity of infection (MOI) of 100 by RAW264.7 macrophages, distinct HslU‐EGFP foci were detected in situ as early as 0.5 h post infection (h.p.i.) (Figure 1B). The percentage of bacterial cells containing HslU‐EGFP foci was 19.7% (Figure 1C). Moreover, bacteria showing HslU‐EGFP foci were also observed in human macrophages differentiated from monocytic leukemia cells (THP‐1) and immortalized murine bone marrow‐derived macrophages (Figure 1C, Figure S1C). Both exponential‐phase and stationary‐phase bacteria formed protein aggresomes upon macrophage infection, confirming that the formation of intracellular bacterial aggresomes is independent of bacterial growth state. In addition, following macrophage phagocytosis, aggresomes labelled by FITC staining are formed in other bacterial species such as *Shigella flexneri* (*S. flexneri*) and *Mycobacterium smegmatis* (*M. smegmatis*) (Figure S1D). Following the invasion of macrophages, *Salmonella* reside in phagosomes, which undergo a series of maturation steps [8]. To further determine the stage of phagosome maturation at which these *Salmonella* form aggresomes, immunofluorescence staining of early endosomes and lysosomes was performed. Phagosomes containing *Salmonella* with aggresomes colocalized with both early endosomes (EEA1) and lysosomes (LAMP1) (Figure S1E), suggesting that gradually matured phagosomes featured by a hostile environment promote the formation of *Salmonella* aggresomes.

Next, C57BL/6 mice were intragastrically inoculated with *Salmonella* whose HslU protein was labeled by acid‐tolerant mECitrine [14]. Immunofluorescence imaging of F4/80 was performed on cryosections of Peyer's patches (PPs) from *Salmonella*‐infected mice, and *Salmonella* with HslU‐mECitrine foci were observed within murine macrophages (Figure 1D). Subsequently, the PPs were lysed, and the released *Salmonella* cells were subjected to microscopy imaging. As early as 2 h.p.i., 29% of *Salmonella* cells in the PPs displayed HslU‐mECitrine foci, and at 120 h.p.i., the proportion of cells with fluorescence foci increased dramatically to 71% (Figure 1E). Whether bacterial aggresomes can form in patients with clinical infectious disease remains unclear and requires further investigation.

### Macrophage‐derived ROS induces the formation of *Salmonella* aggresomes

To elucidate the driving force for the formation of bacterial aggresomes in macrophages, we evaluated the *ex vivo* effect of different phagosomal stress factors [8]. The results revealed that ROS substantially promoted the formation of *Salmonella* aggresomes (Figure 1F), whereas amino acid starvation induced by serine hydroxamate (SHX) and an acidic environment had no effect (Figure S1F,G). Then the ROS stress levels encountered by *Salmonella* were quantitated using the redox‐sensitive biosensor roGFP2 [15]. Following exposure of *Salmonella* to 160 μM menadione, ROS levels in bacterial cells with fluorescently labeled HslU foci were 1.8‐fold higher than those in cells lacking fluorescent foci (Figure 1F,G), suggesting a positive correlation between ROS levels and aggresome formation.

Next, we investigated the relationship between ROS levels and *Salmonella* aggresome formation within macrophages. An increase in CellROX intensity was observed by both flow cytometry and fluorescence imaging after infection, indicating that macrophages generate higher levels of ROS in response to *Salmonella* invasion (Figure 1H,I). We also demonstrated a correlation between the proportion of *Salmonella* exhibiting protein aggresomes (Figure 1C, Figure S1C) and ROS level variations in different macrophage types (Figure S1H,I). Subsequently, we quantified the ROS stress levels encountered by *Salmonella* inside macrophages by using roGFP2 and found that *Salmonella* possessing aggresomes were subjected to a 1.3‐fold higher level of oxidative stress (Figure 1J,K). Moreover, treatment of macrophages with ROS inhibitors reduced the number of aggresome‐positive *Salmonella* (Figure S1J,K). Additionally, C57BL/6 wild‐type and ROS‐deficient (*Cybb* ^−/^^−^) mice were intragastrically inoculated with *Salmonella* to initiate acute systemic infection. Then we detected the percentage of *Salmonella* cells possessing aggresomes released from the spleen and other organs including liver, mesenteric lymph nodes, and PPs. The result illustrated that the proportion of *Salmonella* with aggresome in *Cybb‐/‐* mice was lower than that observed in wild‐type mice at 48 h.p.i. (Figure S1L).

### *Salmonella* aggresomes contribute to macrophage‐induced bacterial antibiotic persistence

**Figure 2:**
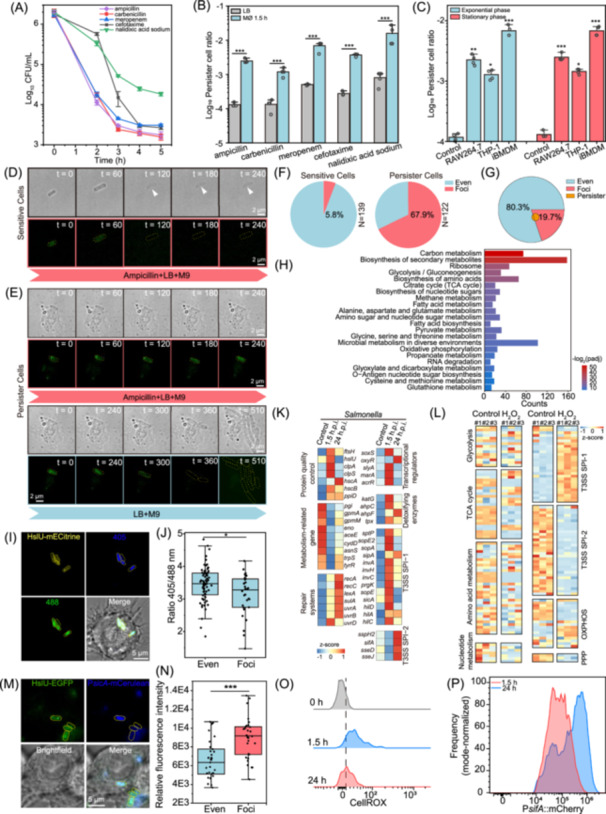
Protein aggresomes within *Salmonella* contribute to macrophage‐induced bacterial antibiotic persistence. (A) Time‐kill curves for *Salmonella* released from RAW264.7 macrophages exposed to 100 μg/mL ampicillin, 100 μg/mL carbenicillin, 5 μg/mL meropenem, 10 μg/mL cefotaxime, and 100 μg/mL nalidixic acid sodium for the indicated lengths of time. (B) Log percentage survival of LB medium‐grown bacteria or bacteria at 1.5 h following phagocytosis by RAW264.7 macrophages after 5 h of treatment with ampicillin, carbenicillin, meropenem, cefotaxime, and nalidixic acid sodium treatment; *n* = 4. (C) Log percentage survival of LB medium‐grown bacteria or bacteria at 1.5 h following phagocytosis by RAW264.7, differentiated THP‐1, and iBMDM cells after 5 h of ampicillin treatment; *n* = 4. (D) Time‐lapse images of *Salmonella* sensitive cells released from macrophages during antibiotic killing; scale bar, 2 μm; *t* = time (min). (E) Time‐lapse images of persister cells with initial HslU‐EGFP foci released from macrophages during antibiotic killing and subsequent resuscitation of surviving cells; scale bar, 2 μm; *t* = time (min). (F) Percentage of aggresome‐containing bacterial cells in different subgroups of *Salmonella* released from macrophages (antibiotic‐sensitive cells and persisters). (G) Contribution of aggresomes to persisters induced by macrophage phagocytosis. Orange denotes persister cells. The orange area in the red zone shows bacteria with protein aggresomes contributing to persister formation, while the orange in the blue zone represents those without aggresomes. (H) KEGG pathway analysis of macrophage phagocytosis‐induced insoluble proteins isolated from *Salmonella*. (I) Live‐cell imaging of *Salmonella* aggresomes and representative QUEEN 7μ_A81D 405ex and 488ex images of *Salmonella* in RAW264.7 cells at 1.5 h.p.i. (scale bar, 5 μm). (J) Average ATP levels in intracellular *Salmonella* with and without aggresomes. (K) Heat map displaying differentially expressed genes in *Salmonella*. (L) Heat map displaying differentially expressed genes between ROS‐treated *Salmonella* and control samples; *n* = 3. (M) Fluorescence images of *sicA* promoter activity in intracellular *Salmonella* (scale bar, 5 μm). (N) Histogram showing *sicA* promoter activity in intracellular bacteria with and without aggresomes. (O) Representative fluorescence‐activated cell sorting (FACS) plots of CellROX in infected macrophages at the indicated time points. (P) Representative FACS plots of mCherry signal from the P*sifA*_mScarlet‐I reporter in *Salmonella* at 1.5 and 24 h.p.i. C was assessed using one‐way ANOVA followed by Fisher's LSD post hoc test; (B), (J), and (N) were assessed using two‐tailed unpaired *t*‐test. Error bars indicate standard deviation; *p < 0.05, **p < 0.01, and ***p < 0.001. KEGG, Kyoto Encyclopedia of Genes and Genomes.

The persister ratio of *Salmonella* internalized by macrophages exhibited a 10‐fold increase compared to uninfected LB‐cultured controls (Figure 2A–C), suggesting bacterial aggresomes may enhance antibiotic persistence. To further verify this point, the bacterial antibiotic killing and resuscitation process was monitored by time‐lapse fluorescence imaging. The result showed that ampicillin (100 μg/mL, 10 × MIC) rapidly lysed *Salmonella* without protein aggresomes, whereas *Salmonella* harboring HslU‐EGFP foci entered a non‐proliferative state and resumed growth in fresh LB medium (Figure 2D,E). Statistical analysis showed that 67.9% of persisters were derived from bacteria with aggresomes (Figure 2F). Considering that only 19.7% of phagocytosed *Salmonella* contained aggresomes (Figure 1C), the probability of persisters originating from cells possessing aggresomes was 8.6‐fold higher than bacteria without aggresomes (Figure 2G).

We then investigated the role of stress‐induced aggresomes and found that ROS‐induced aggresomes also promote persister formation (Figure S2A). Moreover, inhibiting aggresomes with macrophage ROS inhibitors restored antibiotic sensitivity in macrophage‐released *Salmonella* (Figure S2B−D). In vivo, wild‐type and *Cybb^−/−^* mice were intragastrically inoculated with *S*. *Typhimurium* SL1344 and administered with 150 mg/kg cefotaxime intraperitoneally every 12 h. After 48 h, *Salmonella* persister ratio in the organs of *Cybb^−/−^* mice was substantially lower than that in wild‐type mice (Figure S2E).

Given the controversy regarding (p)ppGpp's contribution to persister formation [9, 11], we reexamined its function in macrophage‐induced *Salmonella* persistence. Using Δ*relA*Δ*spoT* ((p)ppGpp synthases), Δ*lon*, and Δ*lon*Δ*sulA* mutants of *S. Typhimurium* SL1344 [16], we found that these mutants showed no substantial decrease in the macrophage‐induced persister cell ratio under ampicillin treatment (Figure S2F). BMDMs were isolated and differentiated by L929 supernatant, with flow cytometry analysis confirming over 90% macrophage purity (CD11b^+^ F4/80^+^) (Figure S2G). We then repeated the persister assay with various antibiotics including ampicillin, carbenicillin, meropenem, cefotaxime, and nalidixic acid sodium. The results also showed a consistent conclusion (Figure S2F,H).

Interestingly, we found that the Δ*lon*, and Δ*lon*Δ*sulA* mutants contained more aggresomes than the wild‐type strain during the 16‐h stationary phase (Figure S2I,J). Meanwhile, the Δ*lon*, and Δ*lon*Δ*sulA* mutants from the stationary phase exhibited higher persister ratios than the wild‐type *Salmonella* under various antibiotic treatments (Figure S2K). We also found that bacterial protein aggresomes formation was effectively suppressed by glucose supplementation, consequently diminishing persister cell formation (Figure S2L,M). In conclusion, our findings offer an alternative explanation that aggresome formation contributes greatly to *Salmonella* antibiotic persistence.

### Aggresomes induced by macrophage phagocytosis facilitate bacterial cell dormancy

To characterize *Salmonella* aggresome composition, we collected *Salmonella* released from macrophages sorted by fluorescence‐activated cell sorting (Figure S3A), and isolated their insoluble proteins. Mass‐spectrometry analysis identified 779 proteins (Table S1), with Kyoto Encyclopedia of Genes and Genomes (KEGG) pathway analysis revealing enrichment in ribosome, carbon metabolism, biosynthesis of amino acids, and oxidative phosphorylation (Figure 2H). Primary sequestered components included ribosomal proteins (rpsA, rplQ, and rplC) impairing translation and amino acid biosynthesis enzymes (asnB, trpB, and gltB) compromising metabolic homeostasis [3, 17]. Comparative proteomics demonstrated consistent aggresome composition across infection, prolonged culture, and ROS stress conditions (Figure S3B,C). Following ROS stress challenge, bulk bacterial ATP levels decreased by nearly 10‐fold (Figure S3D). Since bacteria possessing aggresomes encountered higher ROS levels (Figure 1K), we hypothesized they would have lower ATP concentrations. To investigate single‐cell ATP dynamics, we employed the QUEEN 7μ_A81D biosensor [18], first validating its responsiveness in *Salmonella* through CCCP and 2DG‐induced ATP depletion (Figure S3E). Subsequent in vivo infection assays revealed lower ATP levels in bacterial cells containing aggresomes (Figure 2I,J).

### Intracellular *Salmonella* containing aggresomes exhibit a dormant but SPI‐1 T3SS highly expressing phenotype

To further investigate the interplay between *Salmonella* and host cells, we performed dual RNA sequencing of infected macrophages (Figure S4A and Table S2). Macrophage transcriptomics demonstrated the upregulated expression of host resistance factor‐related genes associated with ROS production, nutritional immunity, autophagy, and inflammasome activation (Figure S4B), which can reduce intracellular *Salmonella* metabolism and restrict bacterial proliferation [19]. *Salmonella* transcriptome showed compensatory upregulation of genes involved in bacterial protein quality control and antioxidant defense system (Figure 2K). In addition, numerous metabolism‐related genes involved in glycolysis, the TCA cycle, oxidative phosphorylation, and amino acid metabolism were suppressed (Figure 2K), suggesting that intracellular bacteria enter a dormant state.

Nevertheless, *Salmonella* can survive and replicate within macrophages according to the expression of type III secretion systems (T3SSs) encoded on *Salmonella* pathogenicity island 1 (SPI‐1) and SPI‐2 [20]. The expression of *Salmonella* SPI‐1 genes increased following phagocytosis by macrophages for 1.5 h compared with that seen in extracellular bacteria (Figure 2K). Moreover, intracellular *Salmonella* with aggresomes exhibited higher SPI‐1‐related promoter activity than *Salmonella* without aggresomes (Figures S4C, Figure 2M,N). RNA‐seq results from *Salmonella* exposed to hydrogen peroxide (H_2_O_2_) stress indicated that ROS serves as a signal to induce bacterial T3SS1 effectors (Figure 2L, Figure S4D−F, and Table S3). After exposure to H_2_O_2_ stress, *Salmonella* possessing aggresomes also exhibited higher fluorescence intensity of SPI‐1 promoters than those lacking aggresomes (Figure S4G–I). Taken together, these findings suggest that *Salmonella* with aggresomes still maintain viability.

### Decreased ROS production by macrophages facilitates the expression of *Salmonella* SPI‐2 effectors and regrowth

Next, we explored under what conditions bacteria with aggresomes can exit dormancy and resuscitate. We investigated the dynamic changes in ROS levels within macrophages at 1.5 and 24 h.p.i. The result of flow cytometry analysis revealed a decay in ROS stress experienced by intracellular *Salmonella* (Figure 2O). Concurrently, we observed an upregulation in the promoter activity of SPI‐2 effectors, including *sifA* and *sspH2*, which facilitated the intracellular proliferation of *Salmonella* (Figure 2P, Figure S4J). In addition, we investigated additional time points and plotted ROS levels against SPI‐2 promoter activity. These results provided stronger correlative evidence regarding the link between reduced ROS production and increased SPI‐2 effector expression (Figure S4K,L).

We hypothesized that the expression of SPI‐2 genes determines the moment at which *Salmonella* possessing aggresomes begin to regrow in macrophages. To verify this assumption, the expression of the T3SS2 *sifA* promoter fused to mScarlet‐I and bacteria resuscitation were monitored in real time. *Salmonella* were phagocytosed by macrophages at an MOI of 5 to ensure that there was only one bacterium per macrophage. When the expression of SPI‐2 genes increased, *Salmonella* aggresomes disassembled, and the bacteria resumed growth and replication (Figure S4M). Taken together, these results revealed that a reduction in ROS stress within macrophages initiates the expression of SPI‐2 genes, accompanied by *Salmonella* aggresome disassembly and later the bacteria regrowth and replication.

## CONCLUSION

In conclusion, our study demonstrates that *Salmonella*‐containing aggresomes released from macrophages evade antibiotic killing by entering a metabolically dormant state, yet retain the ability to resuscitate through regulating virulence gene expression, ultimately enabling recurrent infections.

## AUTHOR CONTRIBUTIONS

**Xiao Chen**: Conceptualization; investigation; writing—original draft; methodology; validation; visualization; writing—review and editing; software; formal analysis; data curation. **Kefan Fang**: Investigation; writing—original draft; methodology; validation; visualization; writing—review and editing; software; formal analysis; data curation. **Bo Li**: Methodology. **Yingxing Li**: Methodology. **Yuehua Ke**: Investigation. **Weixin Ke**: Investigation. **Tian Tian**: Methodology. **Yifan Zhao**: Methodology. **Linqi Wang**: Resources; investigation. **Jing Geng**: Investigation. **Mark C. Leake**: Funding acquisition; writing—original draft; writing—review and editing. **Fan Bai**: Conceptualization; funding acquisition; writing—original draft; writing—review and editing; project administration; supervision; resources.

## CONFLICT OF INTEREST STATEMENT

The authors declare no conflicts of interest.

## Supporting information


**Figure S1:** Fluorescence labeling of bacteria and aggresomes.
**Figure S2:** Correlation between *Salmonella* aggresome formation and bacterial antibiotic persistence.
**Figure S3:** Aggresomes induced by macrophage phagocytosis facilitate bacterial cell dormancy.
**Figure S4:** ROS is an activation signal for *Salmonella* SPI‐1 genes.

**Table S1:** Total insoluble protein mass‐spectrometric analysis.
**Table S2:** Dual RNA‐seq data.
**Table S3:** Bacterial RNA‐seq data.
**Table S4:** Strains, plasmids, and primers used in this study.

## Data Availability

All data supporting the findings of this study are included in the main text and the supplementary materials. The transcriptome data analysis and image processing code utilized in this study have been deposited in the DRYAD repository and are publicly accessible at https://datadryad.org/stash/share/jxa9kd8pdEda_4SDRL2Z811D23I9TQoUr9CLoGErUHc. Supplementary materials (methods, figures, tables, graphical abstract, slides, videos, Chinese translated version and update materials) may be found in the online DOI or iMeta Science http://www.imeta.science/. The data that support the findings of this study are openly available in NCBI at https://www.ncbi.nlm.nih.gov/bioproject/, reference number PRJNA1013683.
