# Supplementary material for: Macrophage‐derived reactive oxygen species promote Salmonella aggresome formation contributing to bacterial antibiotic persistence
Source: Imeta. 2025 Jun 22;4(4):e70059. doi: 10.1002/imt2.70059 (PMC12371251; doi:10.1002/imt2.70059)
Supplement: Supplementary file 1 — Figure S1: Fluorescence labeling of bacteria and aggresomes. Figure S2: Correlation between Salmonella aggresome formation and bacterial antibiotic persistence. Figure S3: Aggresomes induced by macrophage phagocytosis facilitate bacterial cell dormancy. Figure S4: ROS is an activation signal for Salmonella SPI‐1 genes. [file IMT2-4-e70059-s001.docx]

**Supporting information to**

Macrophage-derived reactive oxygen species promote *Salmonella* aggresome formation contributing to bacterial antibiotic persistence

**Running title**: ROS-induced *Salmonella* aggresomes promote intracellular antibiotic persistence

Xiao Chen^1#^, Kefan Fang^1#^, Bo Li^1^, Yingxing Li^2^, Yuehua Ke^3^, Weixin Ke^4^, Tian Tian^1^, Yifan Zhao^1^, Linqi Wang^4,5^, Jing Geng^6^, Mark C. Leake^7,8^, Fan Bai^1^*

^1^ Biomedical Pioneering Innovation Center (BIOPIC), Beijing Advanced Innovation Center for Genomics (ICG), School of Life Sciences, Peking University; Beijing, China.

^2^ Medical Research Center, State Key Laboratory of Complex Severe and Rare Diseases, Peking Union Medical College Hospital, Chinese Academy of Medical Science; Beijing, China.

^3^ Chinese PLA Center for Disease Control and Prevention; Beijing, China.

^4^ State Key Laboratory of Mycology, Institute of Microbiology, Chinese Academy of Sciences; Beijing, China.

^5^ College of Life Sciences, University of Chinese Academy of Sciences; Beijing, China.

^6^ National Local Joint Engineering Research Center of Biodiagnostics and Biotherapy, The Second Affiliated Hospital of Xi' an Jiaotong University; Xi' an, China.

^7^ School of Physics, Engineering and Technology, University of York; York, United Kingdom.

^8^ Department of Biology, University of York; York, United Kingdom.

^#^These authors contributed equally: Xiao Chen, Kefan Fang

*Correspondence: [fbai@pku.edu.cn](mailto:fbai@pku.edu.cn) (Fan Bai)

**Materials and Methods**

**Bacterial strains and plasmids**

The *Salmonella Typhimurium* strains SL1344 and *Shigella flexneri* 2a strain 301 were grown in Luria Broth (LB) medium. *Salmonella* were inoculated into LB medium at a 1:1000 dilution and cultured for 16 hours to reach the stationary phase. Overnight cell cultures were transferred at a ratio of 1:100 into fresh LB medium and cultured for 3 hours to reach the exponential phase. *Mycobacterium smegmatis* strain mc^2^155 was grown in 7H9 broth medium. As required, antibiotics were added at the following concentrations: ampicillin 100 μg/mL, kanamycin 100 μg/mL, and chloramphenicol 25 μg/mL. Bacterial strains, plasmids, and DNA primers are described in Table S4.

**Construction of strains**

Strains harboring target gene-fluorescent protein (FP) translational fusion or single-gene knockout mutants at the chromosomal locus were generated through gene replacement using the λ-red method. The kanamycin resistance gene was amplified from the pKD13 plasmid and introduced via electroporation into bacteria expressing the pSIM6 plasmid containing the λ-red recombinase [1, 2]. The kanamycin resistance gene flanked by frt sites was removed by expressing Flp recombinase using the pCP20 plasmid [1].

**Cell culture**

RAW264.7 macrophages and THP-1 cells were purchased from the Cell Resource Center, Chinese Academy of Medical Sciences and Peking Union Medical College. The iBMDM cell line was a gift from the Zhengfan Jiang lab (School of Life Sciences, Peking University). RAW264.7 and iBMDM cells were cultured in Dulbecco’s modified Eagle’s medium (DMEM) supplemented with 10% fetal calf serum (FCS) at 37℃ in a 5% CO_2_ incubator. THP-1 cells were cultured in Roswell Park Memorial Institute (RPMI) 1640 medium supplemented with 10% fetal calf serum (FCS) at 37℃ in a 5% CO_2_ incubator. To differentiate monocytes into macrophage-like cells, THP-1 cells were cultured in 24-well plates at a density of 1 × 10^5^ cells per well in RPMI supplemented with 200 nM phorbol 12-myristate 13-acetate (PMA; Sigma) for 2 d. Bone marrow-derived macrophages were isolated from C57BL/6 mice and differentiated by supplementation with L929 supernatant.

**Bacterial infection of macrophages**

For infection of mammalian cells, RAW264.7 macrophages were seeded onto 24- well plates (1 × 10^5^ cells/well) and cultured for 24 h at 37℃ in a 5% CO_2_ incubator. The bacterial culture was added to the cells at an appropriate MOI and centrifuged at 800 × *g* for 5 min at room temperature (RT) to synchronize infection. After a 25-min incubation at 37℃, cells were washed three times with PBS to remove extracellular bacteria and incubated in fresh DMEM containing 100 μg/mL gentamycin for 1 h. Subsequently, cells were washed three times with PBS and incubated with fresh DMEM containing 50 μg/mL gentamicin for the remainder of the experiment.

**Mouse infection**

WT C57BL/6 mice were purchased from Beijing Vital River Laboratory Animal Technology. B6.129S-Cybb^tm1Din^/J (*Cybb^-/-^*) mice were obtained from Jackson Laboratory [3]. All mouse experiments were carried out following the National Guidelines for Housing and Care of Laboratory Animals (Ministry of Health, China). The protocol conformed to institutional regulations after review and approval by the Institute of Microbiology, Chinese Academy of Sciences, Beijing. C57BL/6 mice (8 to12 weeks-old) were gavaged with 20 mg of streptomycin (in 200 mL sterile water), and 1 d later were inoculated intragastrically with 2 × 10^8^ *S. Typhimurium* strain SL1344 (in 200 μL of PBS). Bacterial inocula were prepared by 4 h of sub-culture in rich LB medium to reach mid-exponential growth phase prior to gavage. Water was offered immediately, and food was provided 2 h post-infection. Mice were euthanized at 2 h.p.i. and 120 h.p.i.

**FITC staining**

*S. flexneri* and *M. smegmatis* were harvested and washed three times with PBS and incubated with 40 μg/mL FITC for 30 min [4].

**Cell imaging**

After infection, macrophages were fixed in 4% paraformaldehyde (PFA) for 20 min, permeabilized with 0.1% Triton X-100 in PBS (pH 7.4), and then blocked with 1% bovine serum albumin (BSA). Cells were incubated overnight with anti-EEA1 (ab109110, abcam) or anti-LAMP1 antibodies (ab208943, abcam) at 4℃, and incubated the following day with Alexa Fluor 647-conjugated secondary antibodies. The actin cytoskeleton was stained with Alexa Fluor 647-conjugated phalloidin (magenta), and nuclei were stained with DAPI. Immunofluorescence imaging was performed using the DeltaVision OMX SR imaging system in conventional mode (GE Healthcare, USA), which was equipped with a 100 × oil immersion objective (NA 1.49) and EMCCD. Live-cell images were acquired under an inverted microscope (Zeiss Observer Z1, Germany).

**Tissue immunofluorescence imaging**

Mice were transcardially perfused with 10 mL of ice-cold PBS buffer (containing 10 U/mL heparin), followed by 35 mL of 4% PFA in PBS. Perfusion-fixed PPs (following careful removal of intestinal contents), MLNs (following careful removal of fat tissue), and spleen were prepared and post-fixed in 4% PFA overnight at 4°C. The next day, the tissue was dehydrated with 30% sucrose in PBS, snap-frozen, cut into 8-μm sections using a cryostat, and placed on glass slides. Subsequently, slides were dried, fixed in 4% PFA for 20 min, blocked with 2% BSA, and permeabilized with 0.3% Triton X-100 in PBS (pH 7.4). Slides were incubated overnight with anti-F4/80 (14-4801-82, Invitrogen) at 4℃. The next day, slides were rinsed in PBS and incubated with Alexa Fluor 647-conjugated secondary antibodies (1:500) for 1 h (with 5-min staining with DAPI) at RT in the dark.

**Flow cytometry and cell sorting**

Flow cytometry-based analysis was used to measure ROS levels in macrophages. Macrophages were seeded onto six-well plates, cultured for 24 h, and treated with 160 μM menadione or stimulated with the *S. Typhimurium* SL1344 strain. The culture medium was replaced with serum-free DMEM, and CellROX reagent was then added at a final concentration of 5 μM and incubated for 30 min at 37℃. Next, cells were treated with 5% trypsin and resuspended in PBS containing 1% FBS for flow cytometry analysis.

For bacterial cell sorting, Triton X-100 (0.1%) was added to each well and incubated for 20 min at 37°C to selectively lyse macrophages and release the mCherry-expressing *Salmonella*. Electronic noise was excluded using SSC and FSC, and then bacterial samples were sorted according to red fluorescence using a BD FACS Aria SORP Flow Cytometer. Subsequently, the sorted samples were prepared for the persister assay and mass spectrometry. For macrophage cell sorting, infected macrophages were sorted by gating mCherry-positive (mCherry^+^) cells and then collected in tubes containing 20% RNAlater solution diluted in PBS.

**Time-lapse recording of antibiotics killing and subsequent bacterial resuscitation**

The *Salmonella* sorted by FACS were enriched by centrifugation at 15,000 × *g* for 15 min at 4℃. Bacteria were then exposed to 100 μg/mL ampicillin in 90% M9 + 10% LB medium for 4 h at 37℃, which was then replaced with fresh 90% M9 + 10% LB. An FCS2 flow cell chamber system equipped with a temperature controller (Bioptechs) was applied for single-cell observation.

**Insoluble protein isolation and mass spectrometry**

*Salmonella* sorted by FACS after infection were harvested by centrifugation at 5000 × *g* for 15 min at 4°C. The method for insoluble protein isolation from *Salmonella* was modified from Tomoyasu [5]. Pellets were dissolved in 40 mL of buffer A (10 mM potassium phosphate buffer, pH 6.5, 1 mM EDTA, 20% (wt/vol) sucrose, and 1 mg/mL lysozyme) and incubated for 30 min on ice. Cell lysates were mixed with 360 mL of buffer B (10 mM potassium phosphate buffer, pH 6.5, and 1 mM EDTA) and sonication while cooling. The pellet fractions were resuspended in 400 mL of buffer C (buffer B with 2% NP 40) to dissolve membrane proteins. Finally, the aggregated proteins were isolated by centrifugation and re-suspended in 50 mL of buffer B by brief sonication. These isolated proteins were analyzed by mass spectrometry using an Orbitrap Fusion Lumos.

**Dual RNA-seq and data analysis**

Infected macrophages were immediately fixed with 20% RNAlater solution in PBS before FACS. Total RNA was isolated from sorted infected cells using a mirVana kit (Thermo Fisher Scientific). The rRNA was removed using a Ribo-Zero plus rRNA depletion kit (Illumina) according to the manufacturer’s guidelines. cDNA libraries were generated using a VAHTS Universal V8 RNA-seq Library Prep Kit for Illumina (Vazyme). Sequencing was performed using the Illumina HiSeq platform.

Raw data were trimmed with Trimmomatic-0.39 to remove adapters, and the reads were then aligned to the *S. Typhimurium* SL1344 (NCBI RefSeq accession number: NC_016810.1) genome and the mouse (NCBI RefSeq accession number: GCF_000001635.26) genome using HISAT2 (2.2.1). Next, FeatureCounts 2.0.1 was used to generate a table of counts per gene in bacteria or macrophages for each sample for use in downstream differential expression analysis. Differential gene expression was analyzed using R 4.1.0. The “Control” for *Salmonella* infection refers to bacteria incubated in LB medium at 37°C to mid-exponential growth phase. The “Control” for macrophages refers to uninfected macrophages. The Z-score normalized values of genes were calculated for all control and dual RNA-seq samples and heatmaps were plotted using the pheatmap function in R 4.1.0.

**RNA-seq and data analysis**

The bacterial cells were washed twice with normal saline and transferred to 2 mL of normal saline containing 25 mM H_2_O_2_ to induce ROS stress. Bacteria were harvested, and RNA was extracted for further analysis. For transcriptomics analysis, cDNA libraries were generated using the VAHTS Universal V8 RNA-seq Library Prep kit for Illumina (Vazyme). Sequencing was performed using an Illumina HiSeq platform. Sequencing adapter removal and quality-based trimming for raw data were performed using Trimmomatic v. 0.39 with TruSeq3 adapter sequences. Clean data were mapped to the reference genome using HISAT2 (2.2.1). Read numbers mapped to each gene were calculated using featureCounts. Finally, the count from each CDS was normalized to the FPKM value.

**ROS treatment**

Exponential-phase and stationary-phase bacteria from LB cultures were washed twice with 0.9% NaCl and transferred to 2 mL of normal saline containing 25 mM H_2_O_2_ and 0.64 mM menadione, and incubated for 45 min at 37°C.

**Acid-shock treatment**

Exponential-phase and stationary-phase bacteria from LB cultures were collected by centrifugation (4000 × *g* for 2 min at RT). Bacterial pellets were washed twice with 1 mL of PBS, resuspended in fresh acidified (pH 4.5) or neutral (pH 7.2) LB medium, and incubated for 30 min at 37°C.

**SHX treatment**

Exponential-phase and stationary-phase bacteria from LB cultures were pre-exposed to 8 mM serine hydroxamate (SHX) in LB medium for 30 min.

**Persister assay**

Bacteria released from macrophages were diluted 1:20 in fresh LB containing 100 μg/mL ampicillin and then incubated in a shaker (200 rpm) for 5 h at 37℃ before estimating the colony forming unit (CFU) per milliliter. To evaluate the effect of macrophage-produced ROS on *Salmonella* persisters, macrophages were pretreated with 200 μM BHA, 50 μM VAS2870, 1 mM MnTBAP, 1 mM MitoTEMPO, and 5 μM mitoquinone for 3 h before performing the persister assay. There were four replicates.

Wild-type and *Cybb^-/-^* C57BL/6 mice were intragastrically inoculated with 1 × 10^10^ CFU of *S*. *Typhimurium* SL1344 for acute systemic infection. After 2 days, the bacterial load in the spleen, liver, MLNs and PPs of the mice was evaluated. Infected mice were then administered with 150 mg/kg cefotaxime intraperitoneally every 12 h, and bacterial persisters were monitored after 48 h.

**Population-level measurement of bacterial ATP**

Exponential-phase bacteria were washed twice with 0.9% NaCl, and then 25 mM H_2_O_2_ or 0.64 mM menadione was added for 45 min. Bulk ATP levels in the samples were measured using the BacTiter-Glo microbial cell viability assay. Intracellular ATP concentration was determined through the normalization of ATP levels by cell number and single-cell volume [6]. There were three replicates.

**Image analysis**

Analysis of fluorescence imaging data was performed using Python 3.8.16. First, Omnipose 0.4.4 was applied to execute bacterial segmentation to obtain a mask for each bacterium [7]. Subsequently, the presence of protein aggresomes in the bacteria was determined based on the calculation of two fluorescence intensity parameters: maximum fluorescence intensity (fmax) and average fluorescence intensity (a). A bacterium was classified as a protein-aggregated cell if its maximum fluorescence intensity exceeded twice the average fluorescence intensity (fmax > 2a) and if there were a minimum of four adjacent pixels satisfying this criterion [8].

To determine the ATP sensor or ROS sensor ratio, the following steps were performed after characterizing protein aggresomes in each bacterium. First, the fluorescence channels at 405 nm and 488 nm were extracted from each bacterium, and the average fluorescence intensities of these channels were calculated separately. After subtracting the background fluorescence intensity, the ratio of the 405 nm / 488 nm fluorescence intensities in each bacterium was considered as the ROS sensor or ATP sensor ratio.

**Statistical analysis**

Statistical analysis was performed using Origin Pro 2020b, as described in the figure legends. The mean is shown, with error bars indicating standard deviation (SD). The *p* value was calculated using two-tailed unpaired *t*-test or one-way ANOVA followed by Bonferroni/Fisher’s LSD post hoc test; **p* < 0.05, ***p* < 0.01, ****p* < 0.001, and ns, not significant.


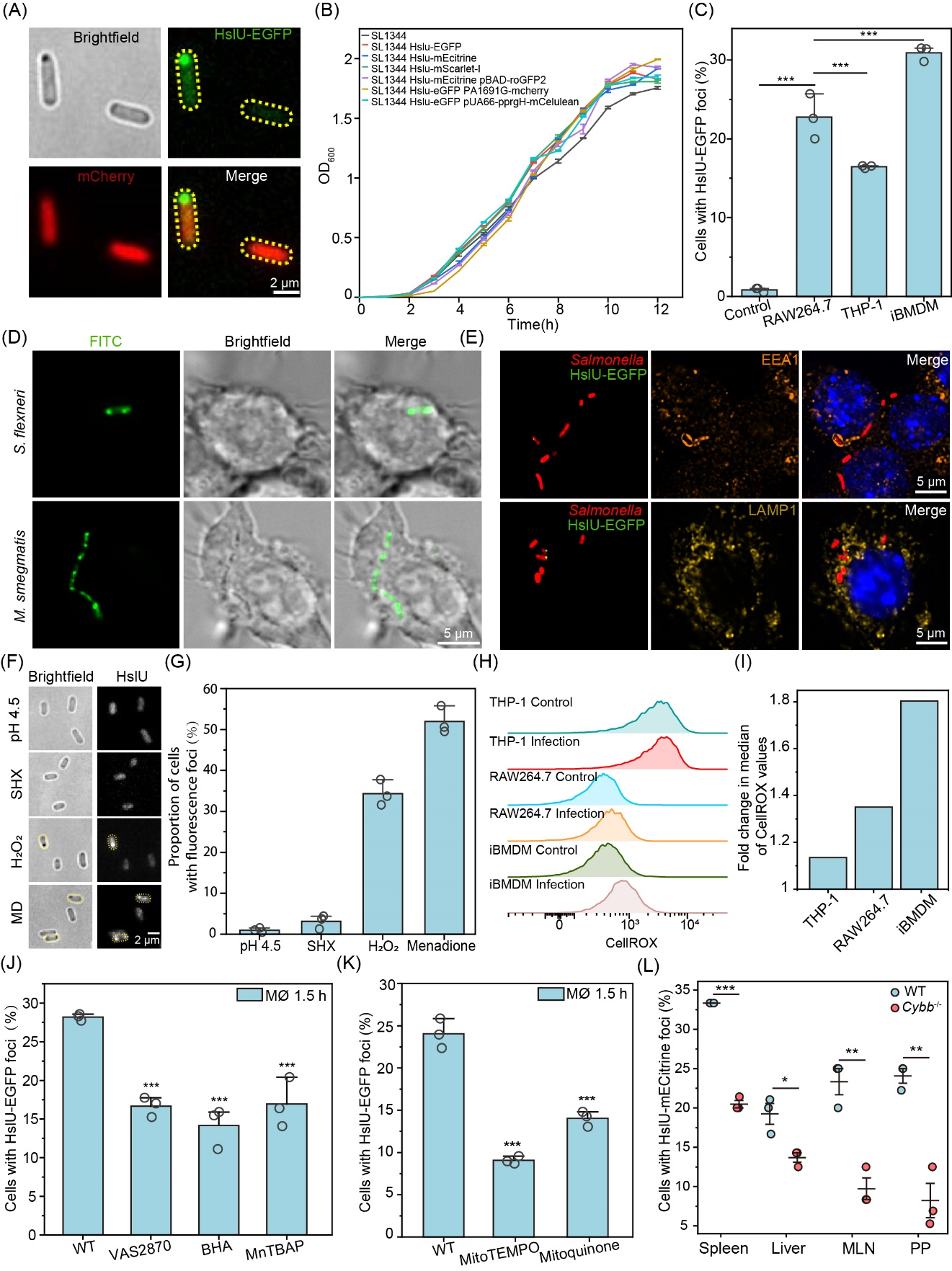


**FIGURE S1** **Fluorescence labeling of bacteria and aggresomes.** (A) Brightfield and fluorescence images of cells labeled with a dual fluorescent protein reporter. *Salmonella* aggresomes were labeled with HslU-EGFP, and the bacterial cytoplasm was labeled with mCherry (scale bar, 2 μm). (B) Growth curves of the strains used in this study. (C) Percentage of *Salmonella* in the exponential phase possessing HslU-EGFP foci after internalization by RAW264.7 macrophages, THP-1 cells, and iBMDM cells; *n* = 3. (D) Live-cell imaging of aggresomes visualized by FITC in *S. flexneri* and *M. smegmatis*, followed by macrophage phagocytosis (scale bar, 5 μm). (E) Immunofluorescence microscopy imaging of *Salmonella* aggresomes in phagosomes. Early endosomes were stained with anti-EEA1 (orange), and late lysosomes were stained with anti-LAMP1 (yellow) (scale bar, 5 μm). (F) Brightfield and fluorescence images of HslU-EGFP/mScarlet-I labeled *Salmonella* cells following treatment with an acidic pH, Serine hydroxamate (SHX), H_2_O_2_, and menadione (scale bar, 2 μm). (G) Percentage of *Salmonella* cells possessing fluorescence foci following treatment with an acidic pH, SHX, H_2_O_2_, and menadione; *n* =3. (H) Representative histograms illustrating CellROX fluorescence. (I) Fold change in median CellROX fluorescence values in differentiated THP-1, RAW264.7, and iBMDM cells following *Salmonella* internalization. (J, K) Percentage of *Salmonella* possessing HslU-EGFP foci after internalization by macrophages in the presence of ROS inhibitors (J) and mitochondrial ROS inhibitors (K); *n* = 3. (L) Percentage of *Salmonella* cells with HslU-mECitrine foci in the spleen, liver, MLNs, and PPs of wild-type and *Cybb^−^*^/−^ mice at 48 h.p.i.; *n* = 3. C, J, and K were assessed using one-way ANOVA followed by Fisher’s LSD post hoc test; L was assessed using two-tailed unpaired *t*-test. Error bars indicate standard deviation; * *p* < 0.05, ** *p* < 0.01, and *** *p* < 0.001.


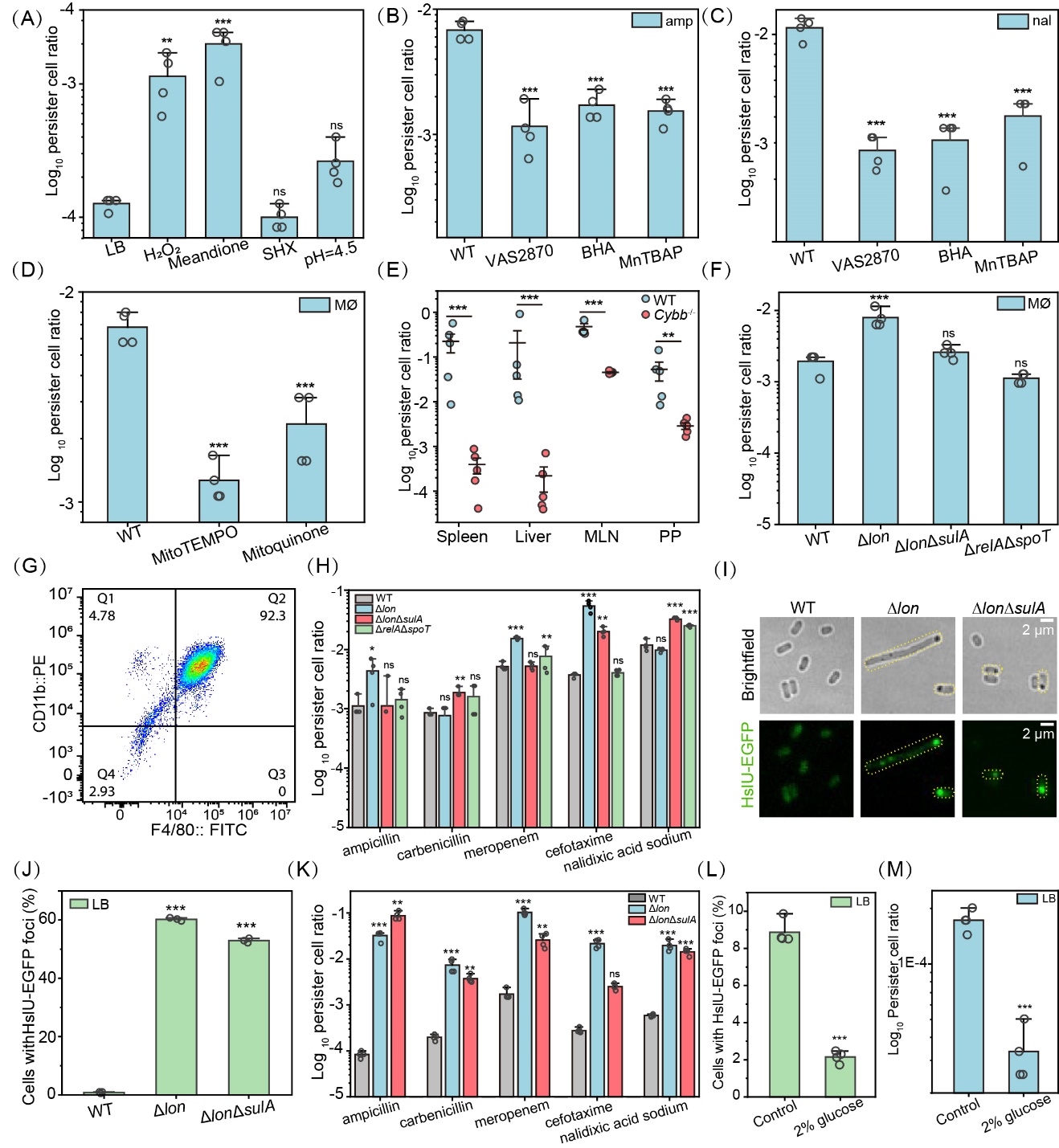


**FIGURE S2 Correlation between *Salmonella* aggresome formation and bacterial antibiotic persistence.** (A) Persister cell ratio of *Salmonella* SL1344 in the stationary phase subjected to an acidic pH, Serine hydroxamate (SHX), H_2_O_2_, and menadione stress; *n* = 4. (B) Persister cell ratio following treatment with 100 μg/mL ampicillin for *Salmonella* phagocytized by macrophages pretreated with ROS inhibitors; *n* = 4. (C) Persister cell ratio following treatment with 100 μg/mL nalidixic acid sodium for *Salmonella* phagocytized by macrophages pretreated with ROS inhibitors; *n* = 4. (D) Persister cell ratio following treatment with 100 μg/mL ampicillin for *Salmonella* phagocytized by macrophages pretreated with mROS inhibitors; *n* = 4. (E) *Salmonella* persister cell ratio recovered from the spleen, liver, MLNs, and PPs of wild-type and *Cybb^−^*^/−^ mice after 48 h of treatment with cefotaxime; *n* = 5. (F) Macrophage-induced persister cell ratio for the SL1344 wild-type and Δ*lon*, Δ*lon*Δ*sulA*, and Δ*relA*Δ*spoT* mutant strains 1.5 h after internalization by RAW264.7 macrophages; *n* = 4. (G) Flow cytometry analysis of BMDMs. Differentiated macrophages were stained with anti-F4/80 FITC and anti-CD11b PE. (H) Macrophage-induced persister cell ratio for the wild-type and Δ*lon*, Δ*lon*Δ*sulA*, and Δ*relA*Δ*spoT* mutant strains 1.5 h after internalization by BMDMs; *n* = 4. (I) Brightfield and fluorescence images of SL1344 wild-type and Δ*lon* and Δ*lon*Δ*sulA* mutant strains during the 16-h stationary phase (scale bar, 2 μm). (J) Percentage of *Salmonella* cells possessing HslU-EGFP foci in the SL1344 wild-type and Δ*lon* and Δ*lon*Δ*sulA* mutant strains; *n* = 3. (K) *Salmonella* persister cell ratio for the SL1344 wild-type and Δ*lon* and Δ*lon*Δ*sulA* mutant strains during the 16-h stationary phase; *n* = 4. (L) Aggresome formation in *Salmonella* SL1344 wild-type strains, with or without 0.2% glucose supplementation, as quantified by the percentage of bacterial cells containing HslU-EGFP foci; *n* = 4. (M) Persister cell frequency in SL1344 wild-type strains, with or without 0.2% glucose supplementation, following treatment with 100 μg/mL ampicillin; *n* = 4. A−D, F, H, J and K were assessed using one-way ANOVA followed by Fisher’s LSD post hoc test; E, L and M was assessed using two-tailed unpaired *t*-test. Error bars indicate standard deviation; * *p* < 0.05, ** *p* < 0.01, and *** *p* < 0.001.


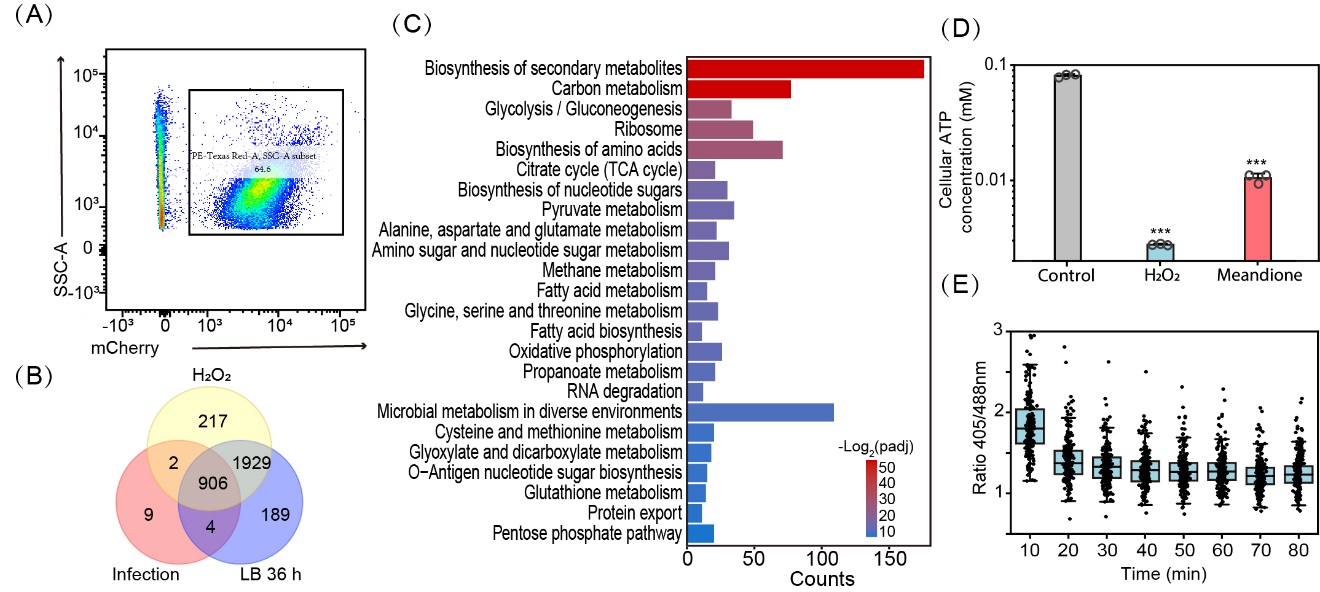


**FIGURE S3** **Aggresomes induced by macrophage phagocytosis facilitate bacterial cell dormancy.** (A) Identification of *Salmonella* in macrophage-derived samples by flow cytometry. Bacterial cells were identified by first gating on events with similar FSC and SSC properties to *Salmonella*, followed by a second gating on particles emitting high levels of red fluorescence (bacterial cells constitutively expressing mCherry). (B, C) Venn diagram (B) and KEGG pathway analysis (C) showing the overlap of insoluble protein expression in bacteria cultured for 36 h in LB medium, subjected to H_2_O_2_ treatment and isolated from macrophage. (D) Cellular ATP concentration in cells treated with 25 mM H_2_O_2_ and 0.64 mM menadione for 45 min; *n* = 3. (E) Changes in the ratio of the QUEEN 7μ_A81D reporter (405 ex/488 ex) per single cell in stationary-phase cultures cotreated with 2-DG and CCCP. D was assessed using one-way ANOVA followed by Fisher’s LSD post hoc test. Error bars indicate standard deviation; *** *p* < 0.001.


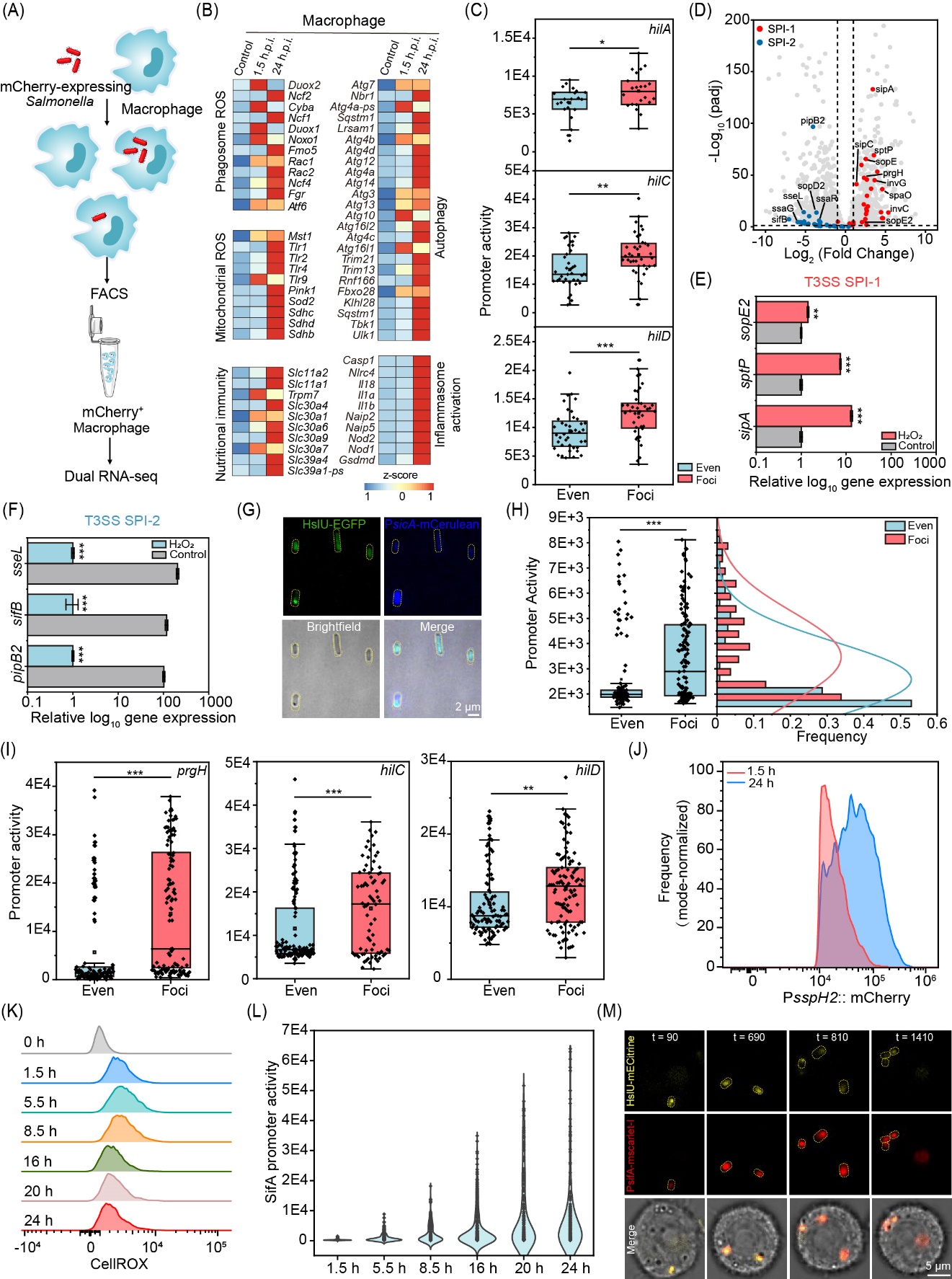


**FIGURE S4 ROS is an activation signal for *Salmonella* SPI-1 genes.** (A) Schematic summarizing the experimental design for dual RNA-seq. (B) Heat map displaying differentially expressed genes in macrophages. (C) SPI-1 T3SS promoter activity of SPI-1, including *hilA*, *hilC*, and *hilD* in intracellular *Salmonella* with and without aggresomes. (D) Volcano plots of differentially expressed mRNA; red dots, representative upregulated SPI-1 T3SS genes; blue dots, representative downregulated SPI-2 T3SS genes. (E, F) Fold increase in mRNA levels of SPI-1 T3SS (E) and SPI-2 T3SS (F) genes following exposure to 25 mM H_2_O_2_ compared with the saline control samples; *n* = 4. (G, H) Images (G) and histogram of *sicA* promoter activity (H) in bacteria following H_2_O_2_ treatment (scale bar, 2 μm). (I) SPI-1 T3SS promoter activity of *prgH*, *hilC*, and *hilD* at the single-cell level in *Salmonella* with and without aggresomes subjected to ROS-mediated stress. (J) Representative FACS plots of mCherry signal from the P*sspH2* mScarlet-I reporter in *Salmonella* at 1.5 and 24 h.p.i. (K) Representative FACS plots of CellROX in infected macrophages at the indicated time points. (L) Promoter activity of *sifA* in intracellular bacteria at the indicated time points. (M) Fluorescence images showing the replication of intracellular *Salmonella* with aggresomes and the promoter activity of *sifA*; scale bar, 5 μm; t = time (min). Two-tailed unpaired *t*-test was used for statistical analyses. Error bars indicate standard deviation; * *p* < 0.05, ** *p* < 0.01, and *** *p* < 0.001.

Reference:

1. Datsenko, Kirill A., Barry L. Wanner. 2000. “One-step inactivation of chromosomal genes in *Escherichia coli* K-12 using PCR products.” *Proceedings of the National Academy of Sciences of the United States of America* 97: 6640−6645. <https://doi.org/10.1073/pnas.120163297>

2. Datta, Simanti, Nina Costantino, Donald L. Court. 2006. “A set of recombineering plasmids for gram-negative bacteria.” *Gene* 379: 109−115. <https://doi.org/10.1016/j.gene.2006.04.018>

3. Matsumoto, Masanori, Seitaro Nakagawa, Lingzhi Zhang, Yuumi Nakamura, Amer E. Villaruz, Michael Otto, Christiane Wolz, Naohiro Inohara, Gabriel Núñez. 2021. “Interaction between *Staphylococcus* Agr virulence and neutrophils regulates pathogen expansion in the skin.” *Cell Host & Microbe* 29: 930−940.e4. <https://doi.org/10.1016/j.chom.2021.03.007>

4. Li, Yanan, Weikang Liang, Chenghua Li. 2023. “Exogenous adenosine and/or guanosine enhances tetracycline sensitivity of persister cells.” *Microbiological Research* 270: 127321. <https://doi.org/10.1016/j.micres.2023.127321>

5. Tomoyasu, Toshifumi, Axel Mogk, Hanno Langen, Pierre Goloubinoff, Bernd Bukau. 2001. “Genetic dissection of the roles of chaperones and proteases in protein folding and degradation in the *Escherichia coli* cytosol.” *Molecular Microbiology* 40: 397−413. <https://doi.org/10.1046/j.1365-2958.2001.02383.x>

6. Conlon, Brian P., Sarah E. Rowe, Autumn Brown Gandt, Austin S. Nuxoll, Niles P. Donegan, Eliza A. Zalis, Geremy Clair, Joshua N. Adkins, Ambrose L. Cheung, Kim Lewis. 2016. “Persister formation in *Staphylococcus aureus* is associated with ATP depletion.” *Nature Microbiology* 18: 16051. <https://doi.org/10.1038/nmicrobiol.2016.51>

7. Cutler, Kevin J., Carsen Stringer, Teresa W. Lo, Luca Rappez, Nicholas Stroustrup, S. Brook Peterson, Paul A. Wiggins, Joseph D. Mougous. 2022. “Omnipose: a high-precision morphology-independent solution for bacterial cell segmentation.” *Nature Methods* 19: 1438−1448. <https://doi.org/10.1038/s41592-022-01639-4>

8. Pu, Yingying, Yingxing Li, Xin Jin, Tian Tian, Qi Ma, Ziyi Zhao, Ssu-Yuan Lin, et al. 2019. “ATP-dependent dynamic protein aggregation regulates bacterial dormancy depth critical for antibiotic tolerance.” *Molecular Cell* 73: 143−156.e4. <https://doi.org/10.1016/j.molcel.2018.10.022>
